# Supplementary material for: Are carbon emissions trading and green financial instruments synergistic? -Comprehensive quantitative research based on content analysis
Source: PLoS One. 2024 Mar 7;19(3):e0298601. doi: 10.1371/journal.pone.0298601 (PMC10919601; doi:10.1371/journal.pone.0298601)
Supplement: S1 Data — (ZIP) [file pone.0298601.s001.zip › Data/地级市Z_F工作报告.zip!/╕╩╦α/╠∞╦«╩╨/└┤╫╘╣1⁄2╓┌║┼í╛┬φ┐╦╩2╛▌═°í┐_2008.docx]

各位代表:

现在,我代表市人民政府向大会作工作报告,请予审议。并请政协委员和列席人员提出意见、建议。

2007年工作回顾

2007年是本届政府的开局之年。一年来,在省委、省政府和市委的正确领导下,市政府团结带领并紧紧依靠全市人民,牢牢把握发展的主动权,积极应对困难和挑战,抢抓机遇,开拓创新,真抓实干,实现了经济快速发展和社会全面进步,圆满完成了市五届人大一次会议确定的各项目标任务。

综合经济实力继续提升。全市实现生产总值196.2亿元,比上年增长11.8%。其中,一、二、三产增加值分别为35.8亿元、73.3亿元和87.1亿元,同比增长8%、11.7%和13.3%。全社会固定资产投资完成111.86亿元,增长40.36%。社会消费品零售总额74.09亿元,增长15.9%。大口径财政收入22.76亿元,同比增长22.36%。城镇居民人均可支配收入8319元,农民人均纯收入1803元,分别增长9.7%和8.3%。

工业经济发展明显加快。实施工业强市战略和振兴装备制造业行动计划,大力实施“10强50户”工程,全市工业经济摆脱了低速徘徊状况,呈现规模扩大、项目扩展、效益提升的良好发展态势。“华天科技”股票成功上市,213电器公司成为“中国机电行业影响力企业100强”。全市规模以上工业企业完成增加值35.45亿元,增长12.5%;实现销售收入63.6亿元,增长16.1%;实现利税7.6亿元,增长41.1%;规模以上装备制造企业增长速度高出全市平均增幅10.8个百分点。企业改制取得新进展,10户已破产企业的各项收尾工作顺利结束,3户企业破产终结,7户企业进入破产程序,2户企业新列入国家政策性破产计划。企业科技进步和创新能力不断增强,列入国家、省级创新型试点企业12家,实施工业技术改造项目23项,完成投资23.55亿元。开发新产品130项,实现新产品销售收入12.5亿元。建成国家级和省级企业技术中心11个,省级企业工程技术中心7个。天水长城中低压电器检测中心晋升为国家级低压电器检测中心。星火数控机床荣获“中国标准创新贡献奖”和“中国名牌产品”称号,9户企业的11个产品获甘肃省名牌产品称号。非公有制经济快速发展,实现增加值64.5亿元,增长12.4%。节能减排和环境保护工作成效明显,万元生产总值综合能耗下降4.3%,城市环境质量不断提升。

新农村建设稳步推进。粮食生产在遭受多种自然灾害的情况下再夺丰收,总产达85.95万吨,较创历史最好水平的上年增产1.3万吨。农业产业化经营步伐明显加快,果品、畜牧、蔬菜等区域特色优势产业规模和效益持续增长。主要畜禽养殖量两位数增长,畜牧业实现产值16.3亿元,同比增长53.34%。果业总产值达到11.1亿元,较上年增加2.3亿元。“花牛”苹果获国家地理标志产品保护认证。蔬菜产值15.8亿元,较上年增加3亿元。龙头企业建设成效显著,续建、新建了具有一定规模的农产品加工企业30家。农业基础设施建设力度进一步加大,新建、恢复各类小型水利工程324处,新增有效灌溉面积1万亩,发展节水灌溉面积2万亩,新修梯田10.52万亩。生态环境建设步伐加快,完成各类造林44.63万亩,沼气建设和后续服务管理工作取得新的进展。新农村建设“示范村、推进村”规划编制工作基本完成,示范村优势产业格局初步形成,村容村貌明显改观。扶贫开发工作稳步推进,实施整村推进项目84个,一批长效性扶贫开发项目得到落实。0.81万农村绝对贫困人口基本解决温饱,6.82万低收入人口稳定解决温饱。劳务经济快速发展,全年输转城乡劳动力70万人次,创收21亿元,比上年增加5.4亿元。农业投入较快增长,项目支持力度明显加大,实施各类农业项目539项,到位资金13.5亿元,增长37.7%。国家循环农业示范市项目、国家绿色农业示范区项目、全膜双垄沟播玉米旱作农业项目开始实施,投资2500万美元的亚行贷款北方旱作农业项目签定了备忘录,投资5亿元的麦积三阳川万亩鲜食葡萄基地开工建设。农村改革不断深化,工作活力进一步增强。农村各项社会事业呈现快速发展势头。

城乡基础设施建设取得新进展。实施交通建设项目1025个,年度完成投资28.8亿元。宝天、天定高速公路、天水军民合用机场和六条战备公路建设进展顺利,通乡通村公路和汽车站点建设进展良好。十大市政基础设施项目和十大开发项目圆满完成当年建设计划,城市绿化和名城保护项目顺利实施。完成城市天然气、城区道路及小巷道整修、公厕建设等公用基础设施建设项目38个,完成投资3.7亿元。实施县城基础设施建设项目39项,县城和小城镇建设步伐加快。土地管理、城市管理、防震防灾、消防、人防工作进一步加强。

项目工作和招商引资成效显著。实施重点建设项目80个,完成投资53.22亿元。全社会固定资产投资突破百亿,高出全省平均增幅14个百分点。争取国家、省上投资项目98个,总投资60.05亿元,到位建设补助资金11.74亿元,创近年新高。实施招商引资项目232项,实到引资23.8亿元。引进国外项目6个,争取国外资金4.35亿元。

商贸旅游业不断壮大。万村千乡市场工程深入推进,城乡市场进一步繁荣,建成社区市场、生产要素市场、乡村集贸市场260处。完成进出口总额9000万美元,增长16%。旅游业发展势头强劲,品牌推介工作成效显著。麦积山景区被评为全国“最具潜力的十大风景名胜区”。百里生态旅游风情线建设全面展开,我市被确定为全省旅游业发展三个中心城市之一。全年接待海内外游客338万人次,旅游综合收入11.32亿元,分别增长22.9%和28.67%。

财税金融工作开创新局面。全市一般预算收入完成7.65亿元,同比增长24.13%。专项资金争取大幅度提高,多渠道争取各类资金16.77亿元。加强财税体制改革力度,强化税收征管,调整优化支出结构,全市财政总支出52.16亿元,同比增长33.07%。以“招行引资”和壮大担保实力为突破口,积极实施金融带动战略,辖区内金融机构贷款增速,经营效益创历史最好水平,全市金融机构本外币各项存贷款余额分别为221.43亿元和93.65亿元,较上年分别增长11.51%和17.39%。信贷投放力度加大,贷款周转使用效率提高,全年累计发放贷款59.27亿元,同比增长38.84%。我市被评为“中国金融生态城市”。

社会事业协调发展。“两基”成果不断巩固,高中教育、高等教育教学质量稳步提升。争取各类教育项目资金2.8亿元,完成中小学D级危房改造8.3万平方米。职业教育发展步伐加快,职教资源得到有效整合,中职招生规模比上年增长10%。教学条件进一步改善,教师队伍建设得到加强,成人教育、幼儿教育和特殊教育全面发展。全市实现了二类城市语言文字达标。科技创新能力继续增强,实施各类科技项目302项,取得科技创新成果262项,13项成果荣获甘肃省科技进步奖。成立六大专业化科技公共服务平台,科技特派员试点工作深入开展。我市再度荣获“全国科技进步先进市”称号。加快特色文化大市建设,积极推进文化体制改革,文化建设呈现新气象。成功举办了伏羲公祭大典暨伏羲文化旅游节、轩辕文化旅游节、女娲文化节和花儿会等重大节会,文化遗产保护、文学艺术创作和“三下乡”活动深入开展,麦积山、水帘洞石窟列入丝绸之路申报世界文化遗产备选点。成功承办了“培训西部教师——崔永元慈善基金”首场拍卖会和全省戏曲青年演员大奖赛。推动实施“文化环保工程”,知识产权保护进一步加强。广播电视“村村通”工程和农村电影“2131工程”有序推进,完成市区有线电视数字化转换4

万户。疾病预防控制、计划免疫等公共卫生事业再上新台阶,市一院晋升三级甲等医院,市二院和市中医院分别晋升三级乙等医院,7家医院住院楼和35个乡镇卫生院建设进展顺利,建成社区卫生服务机构35个。全民健身活动广泛开展,成功承办了全国农民武术大赛暨中国天水伏羲武术大会。全面完成了全年人口和计划生育任务,人口自然增长率控制在6.82‰。认真贯彻党的民族宗教政策,民族地区经济社会全面发展。“双拥”工作和国防后备力量建设进一步加强,我市第三次荣获“全国双拥模范城”称号。人事编制、统计、气象、档案(地方志)、外事侨务、老龄、妇女儿童、残疾人和慈善事业全面进步。

民生状况不断改善。就业与再就业不断推进,新增城镇就业人数3.43万人,实现下岗失业人员再就业1.17万人,城镇登记失业率为3.1%。社会保障体系进一步健全,“两个确保”和“三条保障线”制度全面落实。参加养老、失业、基本医疗等五大保险的职工人数达到56.7万人,各项保险金按时足额发放。全市新型农村合作医疗实现了全覆盖,参合率达87.2%;城镇居民医疗保险覆盖面达到69%;城乡医疗救助发放救助金1048万元,受益群众4.2万人次,城乡居民“看病难”的问题得到缓解;全市113所乡镇卫生院在编人员工资实现财政全额供给;贫困高校生政府助学金制度和生源地助学贷款制度全面落实;解决了14.7万人的饮水安全问题;1.36万户农村沼气建设全面完成;1383公里乡村公路开工建设;超额完成“村村通”电话和农村“户户通电”任务;村干部报酬得到提高;城市居民最低生活保障提标扩面工作全面完成,农村低保工作全面推进;开工建设经济适用房30.5万平方米,实施廉租住房保障2514户。强化市场供应调度和保障,启动副食品价格调节基金平抑市场物价,有效遏制了物价上涨的势头,居民消费价格指数为104.9%,低于全省平均5.5%的物价涨幅。

社会大局和谐稳定。加强社会治安综合治理,依法查处和打击各类违法犯罪活动,全力维护社会稳定,“平安天水”建设成
